# Antibody subclass deficiency accelerates tumorigenesis in genetically engineered mouse models of pancreatic cancer

**DOI:** 10.1172/jci.insight.198489

**Published:** 2026-05-05

**Authors:** Jeremy B. Foote, Sujith Sarvesh, Sameer Al Diffalha, David K. Crossman, Changde Cheng, Myung-Hee Kim, Cherlene Hardy, Julienne L. Carstens, Kyoko Kojima, Bart J. Rose, Christopher A. Klug

**Affiliations:** 1 Department of Microbiology,; 2 Division of Hematology and Oncology, School of Medicine,; 3 Department of Pathology,; 4 Department of Genetics,; 5 Department of Biomedical Informatics and Data Science,; 6 Institute for Cancer Outcomes and Survivorship, and; 7 Department of Surgery, University of Alabama at Birmingham, Birmingham, Alabama, USA.

**Keywords:** Gastroenterology, Immunology, Oncology, B cells, Cancer, Fibrosis

## Abstract

Antibody production by B cells has emerged as an important factor in regulating antitumor immunity with both suppressive and promotive roles in cancer. However, the specific effect of antibody deficiency during development of pancreatic ductal adenocarcinoma (PDAC) has not been explored. To address this question, we crossed the well-established KPC mouse model to mice lacking all circulating immunoglobulin (Ig) due to genetic ablation of both Ig secretion and Ig class switching (KPC-μSAID mice). KPC-μSAID mice exhibited a two-fold acceleration in tumor formation, a two-fold reduction in median survival, and increased liver metastases versus KPC-WT control mice. Immunofluorescence analysis of pancreatic tissues from antibody-sufficient KC- and KPC-WT mice showed that IgG was predominantly localized within the extracellular matrix (ECM). Furthermore, in both KC- and KPC-μSAID mice, ECM density and podoplanin^+^ cancer-associated fibroblasts (CAFs) were significantly reduced. In the KPC-μSAID tumor microenvironment (TME), intratumoral myeloid-derived suppressor cells (MDSC) were also increased, while CD4^+^ and CD8^+^ T cells decreased, relative to tumor-bearing KPC-WT mice, with macrophage exhibiting a mixed polarization phenotype. These findings were recapitulated in antibody subclass–deficient, KPC-AID mice, suggesting a potentially novel function of IgG in suppressing PDAC progression by directly or indirectly regulating pancreatic fibrosis and the density of the ECM.

## Abstract 2

<section data-scroll-anchor=”false” data-testid=”conversation-turn-8” data-turn=”assistant” data-turn-id=”request-WEB:304b9300-5839-4097-8697-d17e5f2ca4a3-3” dir=”auto”> Antibodies like Immunoglobulin G help slow pancreatic cancer by shaping tumor tissue and supporting immunity; without them, tumors grow faster, spread more, and weaken immune defenses. </section>

## Introduction

Infiltrating pancreatic ductal adenocarcinoma (PDAC) is projected to be the third leading cause of cancer death in the United States by 2030, with 5-year overall survival (OS) being ~13% (1)^.^ A number of factors account for the limited success of aggressive chemotherapy or targeting immune checkpoints to enhance OS. PDAC is poorly vascularized and comprised of a dense extracellular matrix (ECM) (2, 3), which limits both drug and cytotoxic CD8^+^ T cell access to the tumor (4). PDAC is also associated with a highly immunosuppressive tumor microenvironment (TME) that includes functionally heterogenous cancer-associated fibroblast (CAF) subsets that are major producers of the ECM (5–8), extensive macrophage and neutrophil infiltrates (9), and CD4^+^ Tregs (10) and B cells (11) leading to establishment of an immunosuppressive TME conducive to tumor progression (12, 13).

Studies in mice have shown that B cells can promote PDAC through secretion of immunosuppressive cytokines including IL-10 and IL-35 (11, 14, 15) and by their ability to enhance M2 macrophage polarization through antibody binding to Fcγ receptors on macrophage (16). Conversely, B cell density and localization within the TME has been correlated with favorable prognosis and significantly extended OS in human PDAC, particularly in cases with evidence of tertiary lymphoid structures (TLS) that suggest in situ antitumor antibody generation (17–20). Despite these observations, understanding of the precise role of antibody in PDAC tumor immunity is unclear.

The focus of our study was to determine the effect of antibody deficiency on pancreatic tumorigenesis. To address this question, we crossed the well-established KPC mouse model (21, 22) with mice containing mature B cells that lacked the ability to secrete antibody of any immunglobulin (Ig) isotype due to homozygous deletions of the gene segment encoding the Ig heavy chain secretory domain (μS^–/–^) and the gene encoding AID, which is responsible for Ig class switching (23).

## Results

### Antibody deficiency accelerates pancreatic tumorigenesis and lethality in KPC mice.

**Figure 1:**
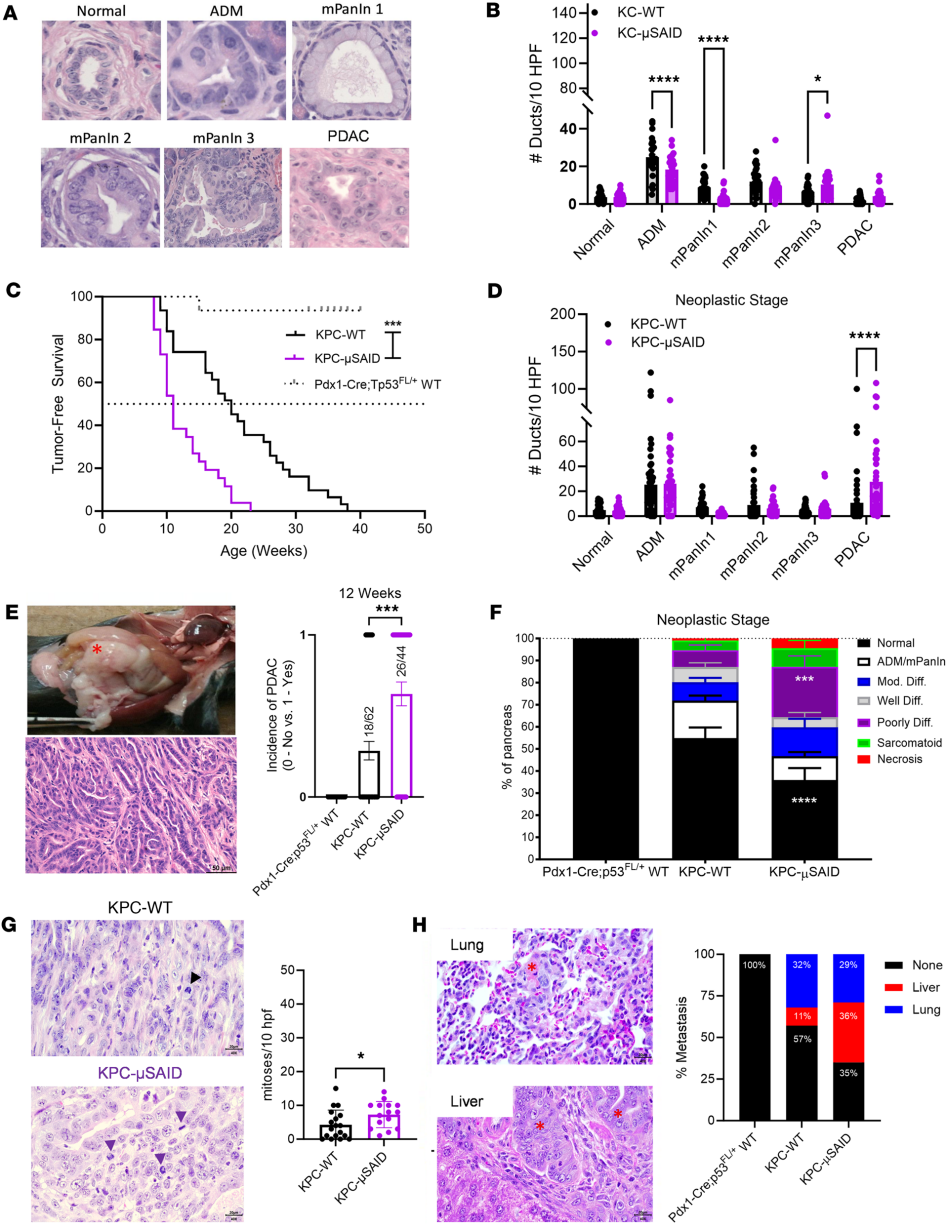
Antibody deficiency accelerates tumorigenesis in the KC and KPC models of pancreatic cancer. (**A**) Representative H&E images of various stages of preinvasive neoplasia, 400X magnification. (**B**) The proportion of normal, acinar-to-ductal metaplasia (ADM), mPanIn1, mPanIn2, and mPanIn3 ducts were assessed in 5-month-old Pdx1-Cre, KC-WT, and KC-μSAID mice by blinded assessment, *n* = 10–34 mice/genotype, *P < 0.05, ****P < 0.0001; 2-way ANOVA with Šídák’s multiple-comparison test. (**C**) Kaplan-Meier survival curve of KPC-WT, KPC-μSAID, and Pdx1-Cre;Tp53^FL/+^ WT control mice, *n* = 23–31 mice/genotype. ***P < 0.001; log rank test. (**D**) The proportion of normal, acinar-to-ductal metaplasia (ADM), mPanIn1, mPanIn2, and mPanIn3 ducts were assessed in 5-month-old Pdx1-Cre, KPC-WT and KPC-μSAID mice by blinded assessment, *n* = 37–52 mice/genotype, ****P < 0.0001; 2-way ANOVA with Šídák’s multiple-comparison test. (**E**) Representative gross image of a pancreatic tumor (left panel, top) that exhibited a well-differentiated phenotype (left panel, bottom). Incidence of PDAC in 12-week-old Pdx1-Cre;Tp53^FL/+^ control, KPC-WT, and KPC-μSAID mice was based on analysis of H&E-stained pancreatic tissue sections, *n* = 10–63 mice/genotype, mean ± SEM, ***P < 0.001; Welch’s *t* test. (**F**) Stacked bar graphs depicting extent of tumor progression in 12-week-old Pdx1-Cre;Tp53^FL/+^ WT control, KPC-WT, and KPC-μSAID mice based on a blinded assessment of the abundance of normal, premalignant (ADM + mPanIn1-3), or locally invasive malignant cells, H&E 40x + 20x, *n* = 30–61 mice/genotype, ***P < 0.001, ****P < 0.0001; mean ± SEM. Two-way ANOVA with Tukey’s multiple-comparison test. (**G**) Left panel: Mitotic figures (arrow) in 10, 40x fields. Right panel: number of mitotic figures were summed from 10 (40×) high-power fields. *P < 0.05; Welch’s *t* test. (**H**) Enhanced liver metastases in KPC-μSAID mice. Left panel: H&E-stained image of lung and liver with intralesional tumor cells (red asterisk) consistent with microscopic metastases (40X). Right panel: Proportions of mice exhibiting microscopic evidence of lung and liver metastases in 12-week-old Pdx1-Cre;Tp53^FL/+^ (*n* = 10), KPC-WT (*n* = 18), and KPC-μSAID mice (*n* = 18) mice. The number mice/lesion is shown as a percentage.

To dissociate the contributions of multiple distinct B cell effector functions from the unique role(s) of antibody in regulating pancreatic cancer progression, we crossed KC- and KPC-WT mice with animals that retain mature B cells that lack the ability to secrete antibody of all Ig isotypes due to deletions of both Ig heavy chain secretory domain (μS) and the gene encoding AID, which is essential for class-switch recombination from IgM to all other Ig isotypes (KC- and KPC-μSAID) (23). Resulting KC- and KPC-μSAID mice lacked all circulating Ig based on serum ELISA (Supplemental Figure 1, A and B; supplemental material available online with this article; https://doi.org/10.1172/jci.insight.198489DS1). Assessment of pathologic changes in pancreata of early-stage, 5-month-old Pdx1-Cre-WT, KC-WT, and KC-μSAID mice revealed a significant decrease in early mPanIn1 (2.7 versus 8.4/10 high-power fields (HPF), *P* < 0.0001), an increase in mPanIn3 (9.9 versus 6.2/10 HPF, *P* = 0.034) in KC-μSAID versus KC-WT mice (Figure 1, A and B), suggesting accelerated preneoplastic progression. Interestingly, the overall area of affected pancreas, which includes the combination of the fibroinflammatory response, acinar-to-ductal metaplasia, and preneoplastic transformation was reduced in 5-month-old KC-μSAID mice (Supplemental Figure 1C). However, evaluation of 10-month-old KC-WT and KC-μSAID pancreata revealed significant differences in pancreatic weights, indicative of greater pancreas involvement resulting from antibody deficiency (Supplemental Figure 1D). These findings indicate delayed involvement of the entire pancreas in this process, despite clear indications of acceleration through preneoplastic stages. Analysis of survival of KC-WT and KC-μSAID mice where tumor progression was the attributed cause of death/euthanasia over a 10-month interval revealed a decrease in survival in antibody-deficient KC-μSAID mice, with KC-μSAID mice exhibiting a median survival of 9.1 months versus KC-WT mice, which did not reach the median survival over the observed 10-month interval (Supplemental Figure 1E). Furthermore, evaluation of available H&E-stained sections of KC-WT (*n* = 3) and KC-μSAID pancreata (*n* = 5) revealed a greater degree of involvement in KC-μSAID, with most tumors appearing as well differentiated (Supplemental Figure 1E). This indicates that, despite reduced area of affected pancreas at 5 months, antibody deficiency accelerated cancer development and lethality in older KC-μSAID mice.

To better understand the roles of antibody in progression to malignant cancer, we next examined the effect of antibody deficiency in KPC mice. KPC-WT mice with a heterozygous floxed, null allele of *Tp53* develop PDAC with complete penetrance, resulting in a median survival of ~5 months (24). At necropsy, gross and histologic evidence of tumor burden is locoregional with invasion in the abdominal region through seeding of tumor cells (carcinomatosis) often resulting in ascites, yet with minimal evidence of metastases to lungs and liver that is consistent with the *Tp53^FL/+^* model (24, 25). Comparatively, median survival of KPC-μSAID mice, where tumor progression was the attributed cause of death/euthanasia, was significantly reduced compared with KPC-WT mice (Figure 1C; 11 versus 22 weeks, respectively; *P* < 0.001). At 12 weeks, KPC-μSAID mice exhibited a significant increase in pancreatic weight compared with KPC-WT mice, implying advanced tumorigenesis (Supplemental Figure 1F). Histological assessment beginning in regions of preneoplasia revealed a 2-fold increase (27.5 versus 10.9/10 HPF; *P* < 0.0001) in numbers of ducts displaying PDAC (Figure 1D). These findings correlated with gross and histological evidence of increased incidence of PDAC in 12-week-old KPC-μSAID versus KPC-WT mice (Figure 1E) ([26/44] 64% versus [18/64] 29%, respectively, *P* = 0.003), as diagnosed by histologic and IHC findings for ductal adenocarcinoma (Supplemental Figure 1G) and sarcomatoid carcinoma (Supplemental Figure 1H). Morphological evaluation of KPC-μSAID tumors revealed more poorly differentiated appearance (Figure 1F) and an increase in mitotic figures (Figure 1G). Evaluation of perineural and intravascular invasion in H&E-stained primary tumors revealed greater incidence of these lesions in KPC-μSAID mice (Supplemental Figure 1, I and J), which was consistent with microscopic evidence of tumor spread on H&E-stained sections of whole liver/lung lobes that indicated a significant increase in liver metastasis in KPC-μSAID versus KPC-WT mice (Figure 1H). Collectively, these findings indicate that circulating antibody has a significant role in suppressing progression of lethal pancreatic cancer in both KC and KPC mouse models.

Since the tumor vasculature is critical to growth and metastatic spread of pancreatic cancer, we next examined the integrity of vessels within regions of neoplasia in KPC-WT and antibody-deficient KPC-μSAID mice through perfusion of mice with high molecular weight (2,000 kDa) FITC-dextran. Mice were injected with 1 mg of FITC-dextran and then after 5 minutes, pancreata were collected to visualize tissue perfusion focusing on regions of neoplasia. Although our sample size was limited, both KPC-WT and KPC-μSAID pancreata demonstrated similar levels of vascular and tissue perfusion (Supplemental Figure 1K) and area of tumor-associated vasculature (Supplemental Figure 1L), suggesting that antibody deficiency did not significantly alter vascular perfusion in KPC mice.

### IgG antibodies are predominantly localized to the pancreatic ECM.

**Figure 2:**
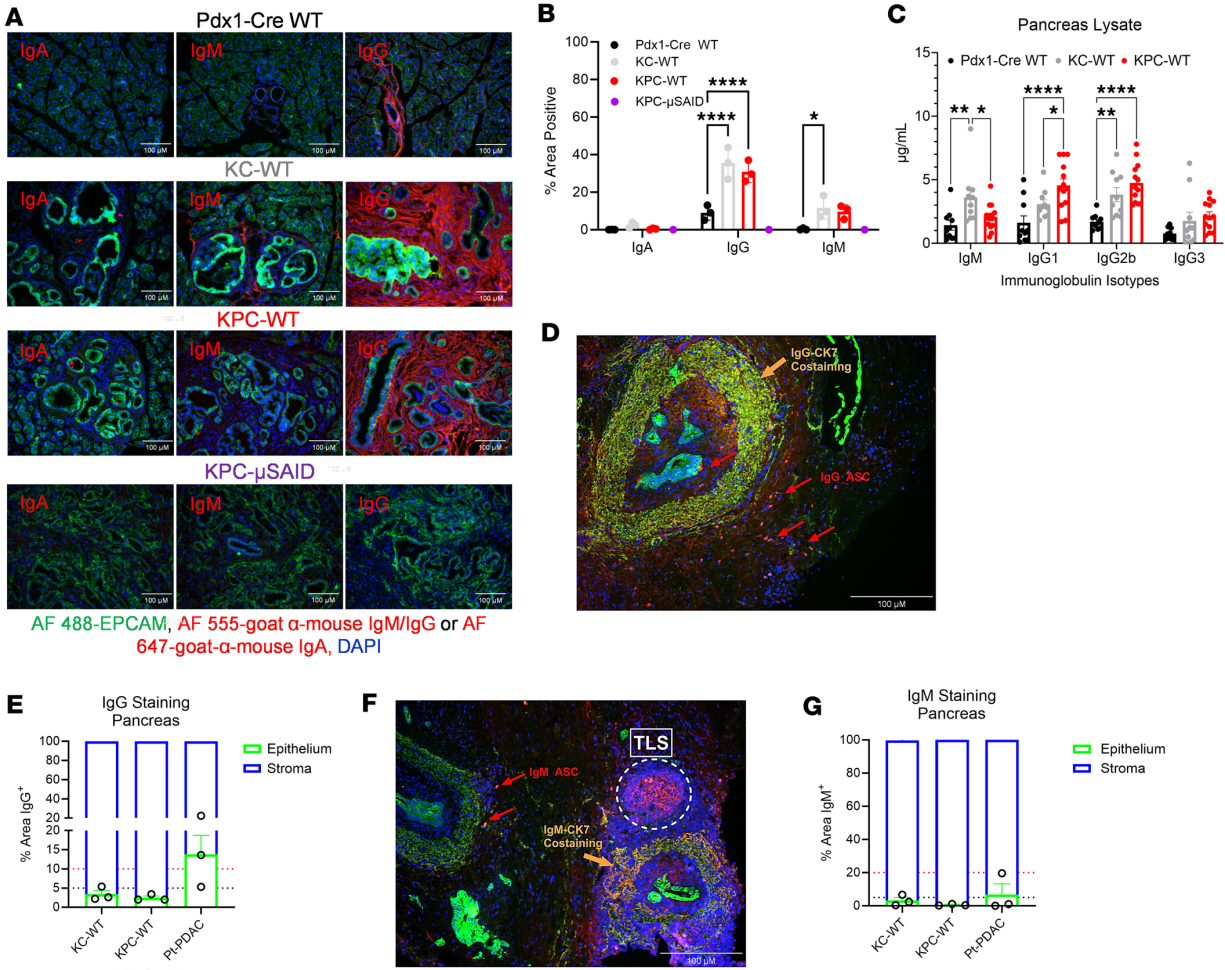
Localization of IgG antibodies to the pancreatic ECM and epithelia of mouse and human PDAC. (**A**) Representative images of IgA, IgM, and IgG staining in Pdx1-Cre WT, KC-WT, KPC-WT, and KPC-μSAID pancreatic tissues. Frozen sections (5 μM) were stained with a cocktail of antibodies that included rat anti-mouse CD326 (Epcam)-AF488, either goat anti-mouse IgM- or IgG-AF555, IgA-AF647, and DAPI. IgA-AF647 was pseudo-colored as “red” in the IgA images to enhance visualization of IgA localization. (**B**) Area of IgA, IgG, and IgM staining in Pdx1-Cre WT (*n* = 3), KC-WT (*n* = 3), KPC-WT (*n* = 3), and KPC-μSAID (*n* = 1) were quantified using ImageJ. Data are graphed as mean ± SD. *P < 0.05, ****P < 0.0001; 2-way ANOVA with Tukey’s correction for multiple comparisons. (**C**) Lysates from homogenized pancreatic tissue from perfused Pdx1-Cre WT, KC-WT, and KPC-WT mice were assessed for mouse IgG1, IgG2b, IgG3, and IgM by ELISA to determine relative abundances of each Ig isotype (*n* = 10–13 mice/group*)*. *P < 0.05, **P < 0.01, and ****P < 0.0001; 2-way ANOVA with Tukey’s correction for multiple comparisons. (**D**) Representative image indicating IgG colocalization (indicated by yellow staining, orange arrow) with selective regions of CK7^+^ tumor tissue in Pt 13 (Supplemental Figure 2). IgG bright cells (presumed antibody-secreting cells, ASC) are highlighted with red arrows. (**E**) Quantification of IgG stain on tumor epithelia versus stroma in KC-WT, KPC-WT, and human PDAC patient tissue. Data are represented as mean ± SD. No statistical significance was reached with the small cohort of samples (*n* = 3/group); Welch’s *t* test. (**F**) IgM staining was proximal to the IgM^+^ tertiary-like lymphoid structure (white-dotted circle). IgM colocalization with tumor epithelia is represented in orange staining region (orange arrow). IgM bright cells (presumed ASCs) are highlighted with red arrows. (**G**) Quantification of IgM stain on tumor epithelia versus stroma in KC-WT, KPC-WT, and human PDAC patient tissue. Data are represented as mean ± SD. No statistical significance was reached with the small cohort of samples (*n* = 3/group); Welch’s *t* test. (**A**, **D**, and **F**) Original magnification, ×16.

Increasing evidence indicates that antitumor antibodies generated in situ in association with TLS within the pancreas may play an important role in antitumor immunity and correlate with significantly prolonged survival in patients (18). To visualize whether recirculating antibodies were localized within the pancreata of perfused KC- or KPC-WT mice, we evaluated coexpression of EpCAM with IgA, IgM, or IgG antibody using immunofluorescence (IF). Consistent with a previous study (26), we observed a significant increase in IgM and IgG in KC-WT and KPC-WT mouse pancreata with predominant localization within the ECM (Figure 2, A and B). Measuring areas of overlapping IgG and EpCAM staining revealed a limited degree of epithelial colocalization at the transition between basement membrane and EpCAM staining for IgG and IgM in KC-WT (% IgG^+^ area, 2.6% ± 1.5%; % IgM^+^ area, 3.2% ± 3.2%) and KPC-WT mice (% IgG^+^ area, 3.5% ± 2.7%; % IgM^+^ area, 0.57% ± 0.47%). ELISA using pancreatic lysates from perfused mice confirmed the IF results, revealing significant increases in IgM, IgG1, and IgG2b antibodies in KC-WT and KPC-WT versus Pdx1-Cre WT mice. Furthermore, with advanced disease, an increase in the presence of IgG1 and IgG2b antibodies in the KPC-WT tumors was noted (Figure 2C). Further analysis will be needed to determine whether IgG localization to areas comprising the ECM is due to specific IgG binding to stromal and/or immune cell types within the ECM, binding to structural features of the ECM like fibronectin (27), or merely the presence of IgG within the tissue space. Since passive diffusion of IgG is limited across endothelial barriers, with antibody actively transported into tissues via transcytosis, it is unlikely that IgG localization within the ECM was largely due to differences in concentration gradients between the serum and tissue, although this remains a formal possibility. Overall, these findings indicate that the vast majority of IgG was localized within the pancreatic ECM and not bound to the neoplastic epithelium in both KC and KPC mouse models of pancreatic cancer.

A previous study measuring antibody binding to pancreatic tumor epithelia in patients revealed ~10% of the IgG detected in the TME was bound to tumor cells (28). To confirm this result, we evaluated IgG staining in the pancreatic TME of a small cohort of patient biopsies (*n* = 3). Similar to findings in mice, IgG antibodies were predominantly localized to the ECM in all PDAC tumor biopsies (Supplemental Figure 2). However, in contrast to mouse tumors, colocalization of both IgM- and IgG-bright antibody-secreting cells was present proximal to specific regions of CK7^+^ tumor epithelia and adjacent tertiary lymphoid-like structures (TLS), with increases in IgG-epithelial staining approximating levels of epithelial-bound IgG in a larger PDAC patient cohort (28) (Figure 2, D and E; 10.5% ± 9.6%). Furthermore, IgM-CK7^+^ colocalization was also present at higher levels than observed in KC-WT and KPC-WT mice (Figures 2, F and G; 25.3% ± 37.9%). Although our human cohort is small, these findings imply a greater degree of antigenicity or recognition of unique tumor antigen(s) in human pancreatic tumors compared with KPC mice (29).

### Antibody deficiency correlates with a reduction in podoplanin^+^ fibroblast and ECM density.

**Figure 3:**
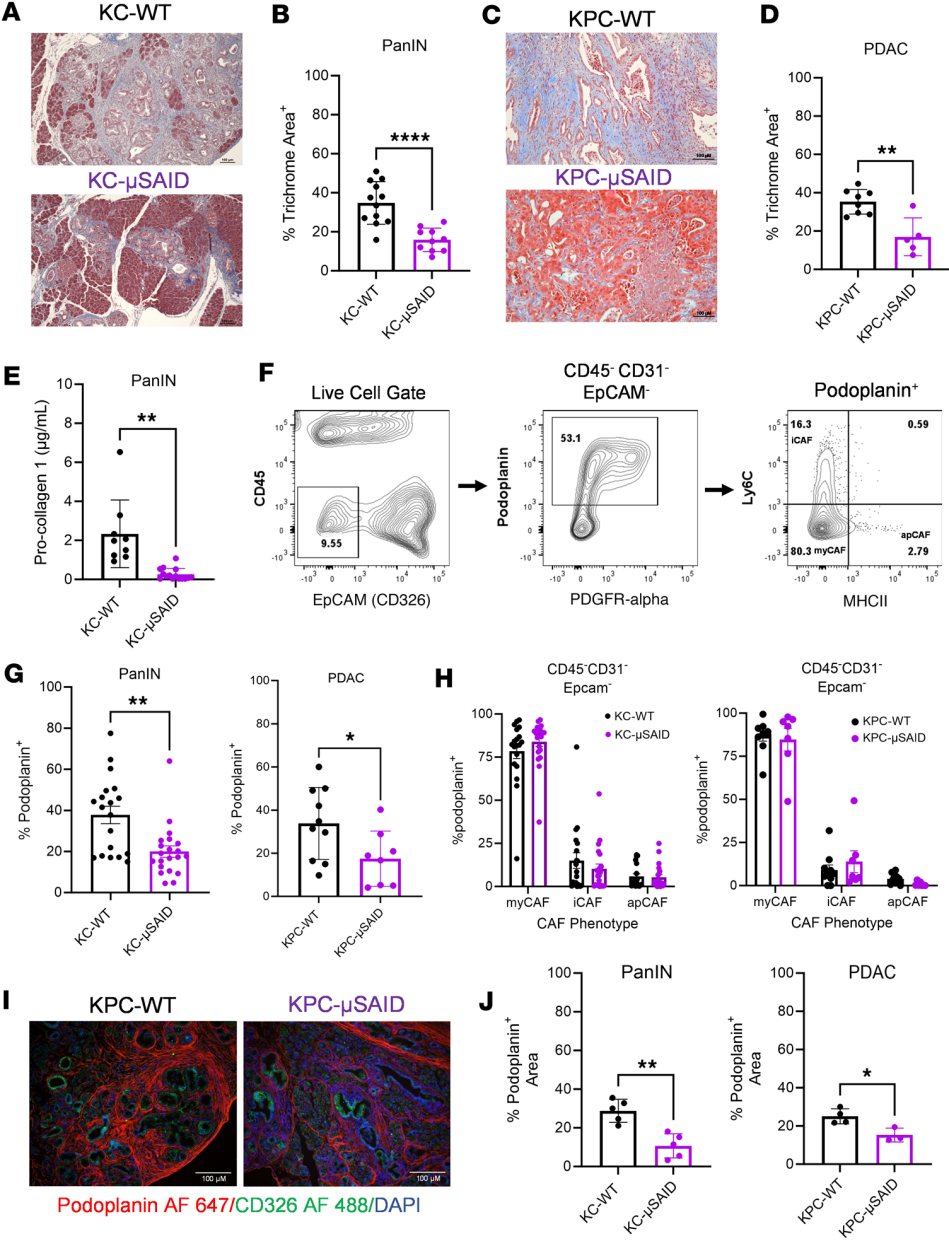
Antibody deficiency reduces ECM and CAF in the KC and KPC pancreatic microenvironment. (**A**–**D**) Masson’s trichrome staining (20× magnification) of pancreas from 20-week-old, mPanIn-bearing, KC-WT and KC-μSAID pancreata (**A**) or 12-week-old, tumor-bearing KPC-WT and KPC-μSAID pancreata (**C**). (**B** and **D**) The area of trichrome staining was assessed using ImageJ software, *n* = 5–12 mice/genotype. Data are representative of aggregated individual experiments, mean ± SEM. **P < 0.01, ****P < 0.0001; Welch’s *t* test. (**E**) Procollagen 1 protein was measured by ELISA using pancreatic lysates from perfused 20-week-old, mPanIn-bearing, KC-WT versus KC-μSAID mice, *n* = 9–15 mice/group, mean ± SD. **P < 0.01; Welch’s *t* test. (**F**) Evaluation of fibroblast abundance in the pancreas of KC-WT and KPC-WT versus KC-μSAID and KPC-μSAID mice using multiparameter flow cytometry. (**G**) Live CD45^–^EpCAM^–^CD31^–^ cells were evaluated for the percentage of cells expressing the pan-cancer-associated fibroblast protein Podoplanin (Pdpn). *P < 0.05, **P < 0.01. (**H**) Podoplanin^+^CD140α^+/–^ CAFs were subsetted into myCAF (Pdpn^+^Ly6C^–^MHCII^–^), iCAF (Pdpn^+^Ly6C^+^MHCII^–^), and apCAF (Pdpn^+^Ly6C^–^MHCII^+^). Data are an aggregate of 5 independent experiments, mean ± SEM. No significant differences were noted between groups; 2-way ANOVA with Šídák’s correction for multiple comparisons. (**I** and **J**) The abundance of podoplanin^+^ CAFs was assessed within the pancreas of KC-WT versus KC-μSAID mice and KPC-WT versus KPC-μSAID mice, *n* = 3–5 mice/group, mean ± SD, *P < 0.05, **P < 0.01; Welch’s *t* test. (**I**) Original magnification, ×20.

Given the striking colocalization of IgG within the ECM, we sought to determine whether there was correlative evidence that this association was meaningful from a biologic perspective. To do this, we evaluated ECM density in KC and KPC pancreata to determine if there was a correlation between ECM density and IgG deposition focusing on regions with ADM, mPanIn, or tumor. Interestingly, antibody-deficient KC-μSAID and KPC-μSAID mice exhibited a 2-fold reduction in the area of trichrome^+^ staining (Figure 3, A–D) within both premalignant regions of ADM/mPanIn and malignant regions of PDAC, consistent with a reduction in procollagen I, a soluble precursor to the most abundant protein within the pancreatic ECM (Figure 3E) (6).

Given that activated myofibroblasts (myCAF) are the predominant cell type responsible for secretion of ECM molecules, we then assessed the abundance of fibroblast subsets using an established flow cytometric panel that utilizes podoplanin as a pan-CAF/fibroblast marker (Figure 3F) (30). The percentage of podoplanin^+^ cells was significantly reduced in KC-μSAID and KPC-μSAID pancreata compared with KC-WT and KPC-WT mice (Figure 3G). However, further subsetting of podoplanin^+^ CAF revealed no significant differences in the relative proportions of myofibroblastic (podoplanin^+^Ly6C^–^MHCII^–^, myCAF), inflammatory (podoplanin^+^Ly6C^+^MHCII^–^, iCAF) and antigen-presenting (podoplanin^+^Ly6C^–^MHCII^hi^, apCAF) CAF (Figure 3H) associated with antibody deficiency. Evaluation of podoplanin expression within affected mPanIn/ADM and PDAC regions revealed a significant decrease in podoplanin expression within regions of preneoplasia/neoplasia in both KC- and KPC-μSAID pancreata compared with KC- and KPC-WT pancreata, respectively (Figure 3, I and J), indicating an effect of antibody deficiency on the density of podoplanin^+^ CAFs within the premalignant and malignant cancer-associated stroma.

To better understand whether IgG antibodies interact with cells in the tumor stroma, we examined expression and localization of IgG with Fcγ receptors. Previous findings indicate that *Fcγr1* and *Fcγr3* mRNA are expressed in podoplanin^+^ CAFs in PDAC (30), suggesting potential for direct antibody-Fc*γ* receptor interactions on CAF subsets. FACS analysis of activating (Fc*γ*R1 and Fc*γ*R3) and inhibitory (Fc*γ*R2b) Fc receptor protein expression on the cell surface of both immune and nonimmune cell types revealed varying levels of Fc*γ*R1/2b/3 on macrophage/mMDSC (high), granulocytic myeloid-derived suppressor cells (gMDSC, intermediate/low), CD31^+^ endothelial, and podoplanin^+^ CAF/fibroblast (low) subsets (Supplemental Figure 3, A and B). Podoplanin^+^ CAFs demonstrated a diffuse pattern of colocalization (Supplemental Figure 3C), whereas F4/80^+^ macrophage were localized proximal to the epithelial basement membrane and showed focal IgG staining (Supplemental Figure 3D). These findings indicated considerable levels of colorimetric (yellow) overlap between these subsets and IgG antibodies in KC-WT and KPC-WT pancreas. Overall, these findings indicate that podoplanin^+^ CAF within the KC and KPC TME predominantly express Fc*γ*R1 protein on the cell surface, while significantly higher levels of both activating (Fc*γ*R1 and Fc*γ*R3) and inhibitory (Fc*γ*R2b) receptors are present on myeloid subsets, in particular monocytes and macrophage. Given the proximity of the IgG antibodies and Fcγ receptors, potential interactions are likely and may be of physiologic relevance to ECM homeostasis in premalignant and malignant pancreas cancer.

### Antibody deficiency alters abundance and phenotypes of immune subsets in the pancreatic microenvironment.

**Figure 4:**
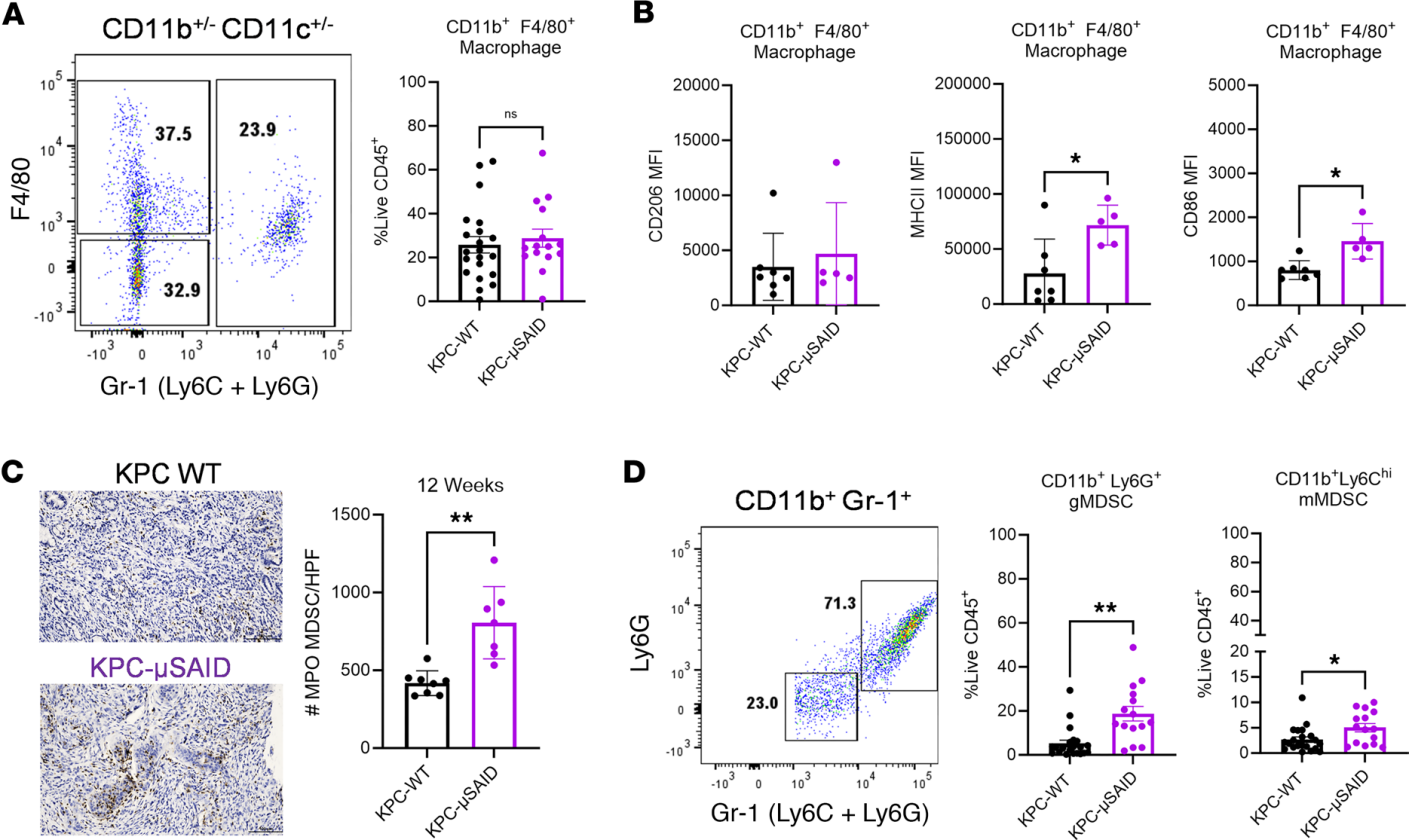
Antibody deficiency alters recruitment and phenotype of myeloid cells in the pancreatic TME. (**A**) Percentages of tumor-infiltrating, CD11b^+^Gr-1^–^F4/80^+^ macrophage in KPC-WT versus KPC-μSAID mice (*n* = 15–21 mice/group), mean ± SEM; Welch’s *t* test. (**B**) Evaluation of coexpression of MHCII, CD206, and CD86 on CD1b^+^Gr-1^–^F4/80^+^ macrophage in tumor-bearing KPC-WT versus KPC-μSAID pancreata, (*n* = 5–7 mice/group) mean ± SD. *P < 0.05; Welch’s *t* test. (**C**) Representative immunohistochemistry for myeloperoxidase (MPO) in pancreata of tumor-bearing, 12-week-old KPC-WT, and KPC-μSAID mice (*n* = 7–8), mean ± SD, **P < 0.01; Welch’s *t* test. Original magnification, ×20. Scale bar, 100 µM. (**D**) Percentages of tumor-infiltrating, CD11b^+^Gr-1^+^Ly6G^+^ gMDSC and CD11b^+^Gr-1^+^Ly6G^–^ mMDSC in KPC-WT versus KPC-μSAID pancreata (*n* = 15–21 mice/group), mean ± SEM, *P < 0.05, **P < 0.01; Welch’s *t* test.

To determine whether the absence of IgG alters the phenotype of myeloid cells, we used multiparameter FACS analysis to assess changes in specific myeloid subsets within the KPC TME. No significant differences in CD11b^+^F4/80^+^ macrophage were noted in KPC-WT and KPC-μSAID mice (Figure 4A); however, antibody deficiency increased the expression of MHCII and CD86 on tumor-infiltrating macrophage (Figure 4B), suggesting an effect on macrophage polarization. Analysis of MDSC subsets within the KPC TME revealed an increase in myeloperoxidase-expressing total MDSCs within the KPC-μSAID TME (Figure 4C), with increases in both Gr-1–expressing granulocytic (Ly6G^+^ gMDSC) and monocytic (Ly6G^–^ MDSC) subsets in tumor-bearing KPC-μSAID mice (Figure 4D). The absence of antibody did not effect the abundance of dendritic cell subsets (Supplemental Figure 4A), including CD11b^–^XCR1^+^ cDC1 (Supplemental Figure 4B) and CD11b^+^CD172α^+^ cDC2 subsets (Supplemental Figure 4C). These findings indicate that antibody deficiency increases the abundance of tumor-infiltrating MDSCs and alters macrophage polarization, indicative of a mixed M1/M2 phenotype.

### Antibody deficiency alters the abundance of T cells in the pancreatic TME.

**Figure 5:**
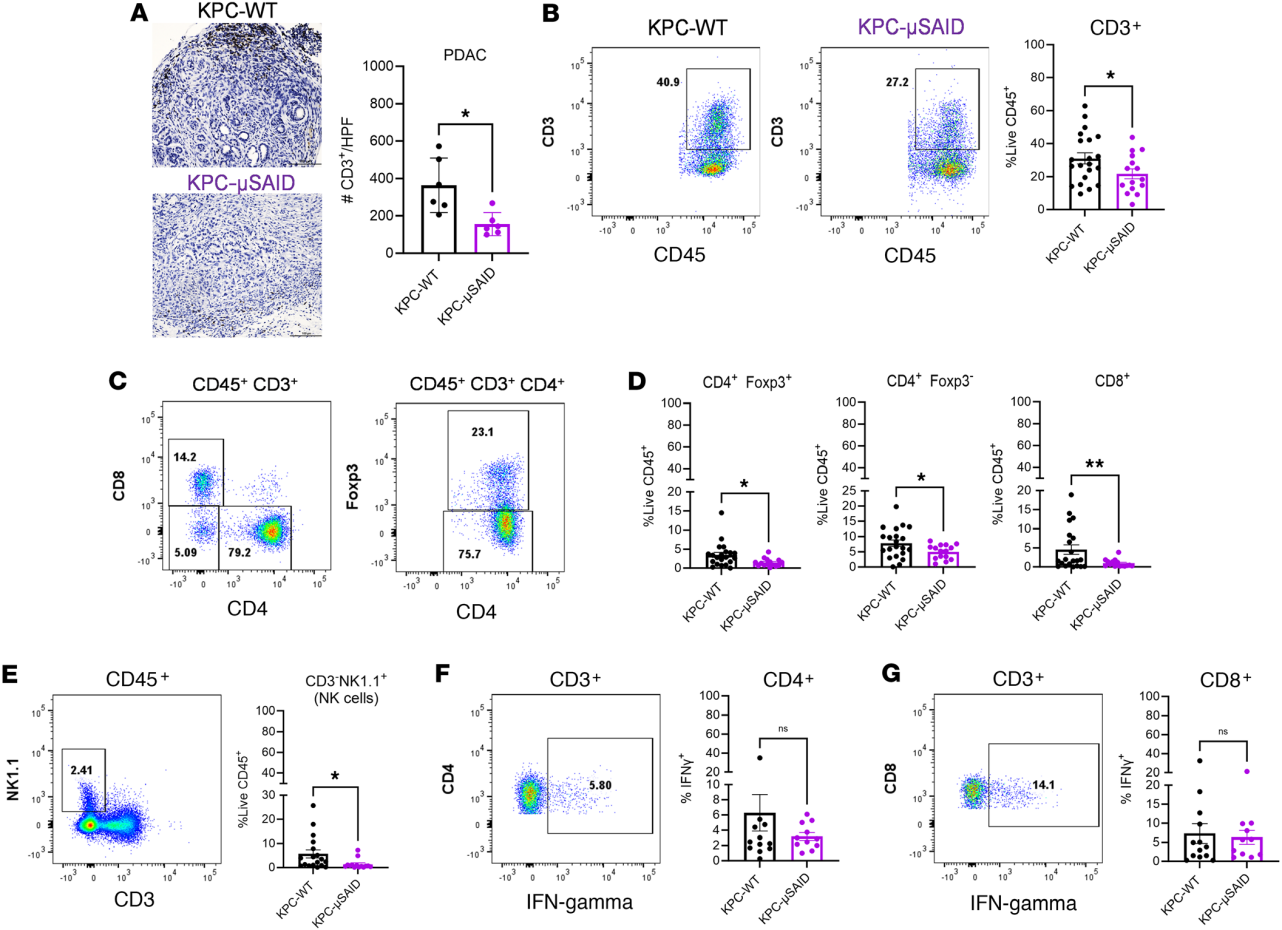
Antibody deficiency alters recruitment of T cell and NK cells in the pancreatic TME. (**A**) IHC for CD3 in tumor-bearing, 12-week-old KPC-WT and KPC-μSAID mice (*n* = 6 mice/group), mean ± SD, *P < 0.05; Welch’s *t* test. Original magnification, ×20. Scale bar, 100 μM**.** (**B**) Percentages of CD3^+^ T cells in KC-WT and KPC-WT versus KC-μSAID and KPC-μSAID mice (*n* = 15–21 mice/group), mean ± SEM, *P < 0.05; Welch’s *t* test. (**C** and **D**) Percentages of tumor-infiltrating CD3^+^CD4^+^Foxp3^+^, CD3^+^CD4^+^Foxp3^–^, and CD3^+^CD8^+^ T cells in 12-week-old KPC-WT and KPC-μSAID pancreata (*n* = 15–21 mice/group), mean ± SD, *P < 0.05, **P < 0.01; Welch’s *t* test. (**E**) Percentages of tumor-infiltrating CD3^–^NK1.1^+^ NK cells in 12-week-old KPC-WT and KPC-μSAID pancreata (*n* = 14–18 mice/group), mean ± SD, *P < 0.05; Welch’s *t* test. (**F** and **G**) Expression of intracytoplasmic IFNγ in CD4^+^ (**F**) and CD8^+^ (**G**) T cells 5 hours after PMA-ionomycin stimulation in the presence of brefeldin A using cells isolated from 12-week-old KPC-WT and KPC-μSAID pancreata (*n* = 11–14 mice/group), mean ± SD, ns = nonsignificant; Welch’s *t* test.

The increased presence of potentially immunosuppressive MDSC subsets in the KPC-μSAID TME, which act as a reservoir for immunosuppressive tumor-associated macrophage (9, 12, 31) and can effect T and NK cell infiltration and function (32–34), suggests that these changes may influence antitumor immunity. Therefore, we evaluated T and NK cell infiltrate and function in the KPC-WT versus the KPC-μSAID TME. Antibody deficiency significantly reduced the proportions of total CD3^+^ T cells in tumor-bearing KPC-μSAID mice (Figure 5, A and B), with the majority of T cells distributed in the peritumoral stroma within lymphoid aggregates (Figure 5A). Within the CD3^+^ T cell subsets, significant reductions were noted in CD4^+^ and CD8^+^ T cells (Figure 5, C–E). Similar to adaptive CD4^+^ and CD8^+^ T cell subsets, CD3^–^NK1.1^+^ NK cells exhibited a decrease in abundance in antibody-deficient KPC-μSAID mice (Figure 5E). Analysis of cytokine production after PMA-ionomycin stimulation revealed no significant differences in IFN-γ secretion from CD4^+^ and CD8^+^ T cells (Figure 5, F and G) isolated from the KPC-WT or KPC-μSAID TME. These findings suggest a reduction in T and NK cell recruitment secondary to increases in MDSC influx into the TME of antibody-deficient KPC-μSAID mice.

### Antibody subclass deficiency mediates the tumor-suppressive function of antibody in PDAC and the density of pancreatic fibrosis.

**Figure 6:**
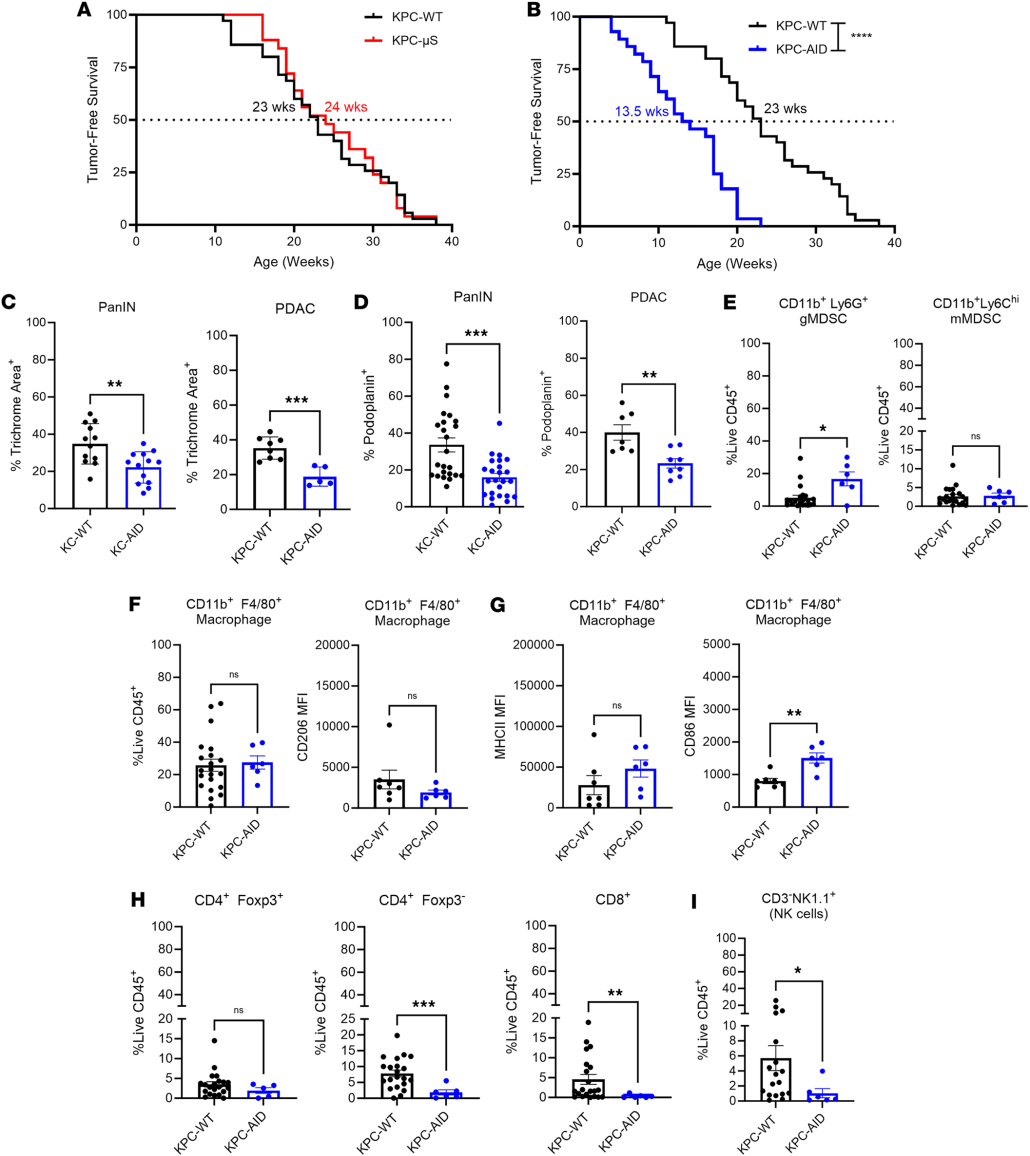
Class-switched antibody (IgA, IgE, IgG) deficiency accelerates pancreatic tumorigenesis and remodels the tumor microenvironment. (**A** and **B**) Kaplan-Meier survival curve of KPC-WT versus KPC-μS (**A**), and KPC-WT versus KPC-AID mice (**B**), *n* = 25–35 mice/genotype, ****P < 0.0001; log rank test. (**C**) The area of trichrome staining was assessed using ImageJ software, *n* = 5–13 mice/genotype. Data are representative of aggregated individual experiments, mean ± SEM, **P < 0.01, ***P < 0.001; Welch’s *t* test. (**D**) Live CD45^–^EPCAM^–^CD31^–^ cells were evaluated for the percentage of cells expressing the pan-cancer-associated fibroblast marker Podoplanin (Pdpn), *n* = 7–24 mice/genotype. Data are representative of aggregated individual experiments, mean ± SEM, **P < 0.01, ***P < 0.001; Welch’s *t* test. (**E**) Percentages of tumor-infiltrating, CD11b^+^Gr-1^+^Ly6G^+^ gMDSC and CD11b^+^Gr-1^+^Ly6G^–^ mMDSC in KPC-WT versus KPC-AID pancreata (*n* = 6–21 mice/group), mean ± SEM, *P < 0.05; Welch’s *t* test. (**F**) Percentages of tumor-infiltrating, CD11b^+^Gr-1^–^F4/80^+^ macrophage in KPC-WT versus KPC-AID mice (*n* = 6–21 mice/group), mean ± SEM; Welch’s *t* test. (**G**) Evaluation of coexpression of CD206, MHCII, and CD86 on CD11b^+^Gr-1^–^F4/80^+^ macrophage in tumor-bearing KPC-WT versus KPC-AID (*n* = 6–7 mice/group) pancreata, mean ± SD, **P < 0.01; Welch’s *t* test. (**H**) Percentages of tumor-infiltrating CD3^+^CD4^+^Foxp3^+^, CD3^+^CD4^+^Foxp3^–^, and CD3^+^CD8^+^ T cells in 12-week-old KPC-WT and KPC-AID pancreata (*n* = 5–21 mice/group), mean ± SD, **P < 0.01, ***P < 0.001; Welch’s *t* test. (**I**) Percentages of tumor-infiltrating CD3^–^NK1.1^+^ NK cells in 12-week-old KPC-WT and KPC-AID pancreata (*n* = 6–18 mice/group), mean ± SD, *P < 0.05; Welch’s *t* test.

Given the predominant localization of IgG and IgM antibodies (and not IgA) in the pancreatic ECM (Figure 2B), we then evaluated the specific effect of deleting only secretory IgM (μS^–/–^) or all class-switched (IgA, IgE, IgG) antibody isotypes (AID^–/–^) on pancreatic tumorigenesis. While KPC-μS–deficient mice did not exhibit differences in median survival compared with KPC-WT mice (Figure 6A), tumor-free and median survival of KPC-AID mice phenocopied the incidence of lethal PDAC observed in KPC-μSAID mice (Figure 6B). This underscores the importance of class-switched antibodies (likely IgG subclass antibodies) in suppression of pancreatic cancer. Furthermore, KPC-AID–deficient animals exhibited similar reductions in ECM (Figure 6C), reductions in podoplanin^+^ CAFs (Figure 6D), increases in gMDSC infiltration (Figure 6E), a similar shift toward M1 macrophage polarization (Figure 6, F and G), and reductions in T and NK cells within the KPC-AID TME (Figure 6, H and I). These findings largely phenocopy our findings with complete antibody-deficient KPC-μSAID mice, indicating a predominant role of IgG and not IgM antibodies in regulation of pancreatic cancer progression and fibrosis.

## Discussion

An unanswered question in the development of pancreatic cancer is whether there is an antitumor antibody response that affects tumor progression and how this shapes the overall immune response at both early and late stages of disease. We addressed this question by analyzing KC and KPC mice with B lymphocytes incapable of generating secreted antibody of any Ig isotype or mice lacking all class-switched antibodies (IgA, IgE, and IgG isotypes). The results show that specific loss of class-switched antibodies, and not IgM, significantly accelerated tumor progression from premalignancy to lethal metastatic ductal adenocarcinoma. The acceleration of metastatic disease was correlated with several potentially important observations that highlight the multifaceted function of antibody in systemic immunity and potentially novel roles in fibrosis. First, we noted that tumors exhibited a tendency for poorly differentiated and sarcomatoid carcinomas, which in patients and mouse models carry poorer overall prognoses with local invasion and metastases (35, 36). This was consistent with enhanced perineural and perivascular localization of tumor epithelial cells in the pancreas, consistent with increased frequency of microscopic metastases (37). Furthermore, endothelial cell function and permeability can be modulated by IgG, likely by IgG binding to activating and inhibitory Fcγ receptors on the surface of the endothelium (38), which may influence tumor cell invasion within the TME. Studies of IgG binding and its effect on endothelial cell function suggested a normal homeostatic role in reducing permeability (39). However, our analysis of whether vascular leakage was affected in the absence of circulating antibody using an in vivo, FITC-dextran permeability assay revealed minimal alteration in leakage of FITC-dextran into the ECM in KPC-WT and KPC-μSAID mice (Supplemental Figure 1K) or differences in the percent area of CD31 vasculature (Supplemental Figure 1L). This suggests that vascular organization within the KPC TME was not affected by antibody deficiency.

Second, loss of circulating class-switched Ig resulted in multiple changes in immune cell infiltration and phenotypes in ways that would be predicted to promote disease progression. Perhaps most significant were the changes in macrophage phenotype with intratumoral F4/80^+^ cells coexpressing CD206, MHCII, and CD86 at higher levels than observed in macrophage from KPC-WT pancreata. IgG-FcγR1 signaling through Btk in combination with FcγR-independent PI3Kγ signaling promotes classical M2 macrophage polarization, immune suppression, and tumor progression in PDAC (16). Colocalization of IgG and FcγR1 on podoplanin^+^ CAF and F4/80^+^ macrophage in the KC-WT and KPC-WT pancreas confirmed proximity of these cells to IgG antibodies, furthering the likelihood of significant interactions occurring in the affected pancreas. Furthermore, significant increases in myeloid-derived suppressor cell infiltration were noted in the KPC-μSAID TME, which are likely contributing to inhibition of T and NK cell infiltration (12, 32–34). Overall, these findings are consistent with hallmarks of an immunologically cold, immune-excluded TME, which is the most common immune subtype seen in patient tumor biopsies that is associated with both increased disease progression and reduced patient survival (40, 41).

An important consideration that was not examined in this work is the effect of antibody subclass deficiency on microbial dysbiosis. IgA antibodies in the intestinal mucosa have a direct effect on microbial communities in the small and large intestine (42), with IgA deficiencies leading to systemic circulation of bacterial species enhancing systemic inflammation (43). Similar to a wide range of disease processes, microbial dysbiosis is a risk factor for pancreatic cancer through promotion of pancreatic inflammation. Correlative studies in patients have provided predictive microbiome signatures associated with increased incidence of PDAC (44–46). Preclinical studies have also demonstrated a correlation between certain flagellated bacterial species and promotion of immune suppressive macrophage phenotypes within the PDAC TME (47). In our study, we noted rare multifocal localization of IgA antibodies proximal to or within the pancreatic ducts, which could imply interactions with organisms translocating into the pancreas or those associated with resident microbiota. Further studies are necessary to evaluate the specific role IgA on the intestinal microbiota and pancreatic cancer progression.

Third, and perhaps most interestingly, the loss of circulating, class-switched antibody resulted in a significant reduction in ECM density and reduced numbers of podoplanin^+^ CAF in the TME. Previous studies targeting depletion of collagen-rich ECM either through genetic (36) or pharmacologic (3, 48) approaches in mice and patients with PDAC, or differences in ECM content occurring naturally within patients (49), demonstrate that a reduction in the ECM correlates with more poorly differentiated tumors and increased metastases. Analysis of tumor biology through single-cell RNA-seq of patient samples with naturally occurring loose versus dense ECM revealed a similar relationship between the absence of dense stroma and tumor progression, with intratumoral increases in a metabolically active CAF subset associated with low desmoplasia and a higher rate of metastasis (49). Loose-type ECM was also associated with a tumor-cell subset expressing transcriptional programs linked to ECM degradation and enhanced metastasis (49).

Although very little binding of IgG or IgM to tumor epithelia was noted in KPC-WT mice, it is clear that in situ T and B cell responses within the PDAC TME, supported by formation of tertiary-like lymphoid tissue, confers a positive survival prognosis in PDAC (median OS of 26.3 versus 14.4 months in cases with and without histologic evidence of TLS, respectively) (18). This implies a role for tumor-antigen-specific antibody responses that support antitumor immunity (17, 18, 20). Previous studies have shown that the KPC GEM models possess very limited neoantigenic epitopes compared with PDAC patient samples (29, 50), with some patient tumor tissues demonstrating direct tumor-epithelial binding of antibodies (28). Using a cohort of 34 PDAC patient biopsies, tumor epithelial cell-bound IgG was ~10% of total IgG in the TME (28). We see a similar extent of tumor epithelial cell binding in our small cohort of patients.

The question of how IgG may be modulating ECM density in PDAC and whether the extensive localization of IgG to the ECM/stroma is a direct biological response to suppress tumor spread or merely trapping of IgG that has diffused through the tumor-associated vasculature requires further investigation. It is also possible that IgG binding within the tumor ECM is functioning in response to a perceived “wound,” as IgG localization to the ECM has also been noted during wound healing (51). In this context, IgG binding may enhance stability and/or assembly of ECM components or function to recruit immunomodulatory cells to damaged tissue. Alternatively, IgG localization within the stroma/ECM could be in response to direct IgG-Fcγ receptor engagement on stromal and immune cells localized within the ECM (16, 26). Our analyses and the work of others (16) has shown high Fcγ receptor expression on monocytes, macrophage, and neutrophils, in addition to modest expression of FcγR1 on podoplanin^+^ CAF. While the significance and diversity of biologic processes initiated by FcγR binding to IgG within innate and adaptive immune subsets is more clearly delineated, very little is known regarding the biologic significance of IgG-FcγR interactions on mesenchymal cells. Previous studies in cardiac fibroblasts reveal a role for FcγR1 signaling in development of myofibroblasts (52), which implies a potential role for FcγR signaling in fibroblast development and function, although mechanistic insight is lacking.

Overall, our findings genetically demonstrate the critical importance of class-switched antibodies in suppression of tumor progression through a potentially novel mechanism that may involve regulation of ECM density. Further studies are needed to determine whether IgG can directly or indirectly influence crosstalk between macrophage and distinct intratumoral CAF subpopulations that result in alterations to ECM density, accumulations of immunosuppressive MDSC, and exclusion of T and NK cells from the TME.

## Methods

### Sex as a biologic variable

Male and female littermate mice of the appropriate genotypes obtained from the breeder cages were utilized in these studies without regard to sex. There was no observable difference between numbers of viable males or female borne in all cohorts, nor was there variability in the kinetics of disease progression associated with loss of circulating antibody. Large cohorts of male and female mice were used in most all analyses to minimize sex bias in the results, which were highly consistent independently of sex.

### Mouse husbandry

Mouse strains *Pdx1-Cre* transgenic (*B6.FVB-Tg(Pdx1-Cre)6Tuv/Nci*) (PMID: 14706336), *LSL-Kras*^G12D/+^ (*B6.129-Kras^tm4Tyj/Nci^*) (PMID: 11751630), and *Tp53*-floxed (*Trp53^tm1Brn^*) (PMID: 11694875) were obtained from the Mouse Models of Human Cancer Consortium (NCI-Frederick) and backcrossed onto a C57BL/6J background for at least 11 generations prior to crossing onto antibody-deficient C57BL/6 *μS^–/–^* *x AICDA^–/–^* (*AID^–/–^*) double-knockout (*μSAID^–/–^*), antibody subclass-deficient *AID^–/–^* knockout, or secretory IgM-deficient *μS^–/–^* mice (all on the C57BL/6 background). *μSAID* double-knockout mice (23) were obtained from Frances Lund at UAB. KC and KPC mice were analyzed as models of premalignancy (KC) and frank adenocarcinoma/PDAC (KPC). Mice were maintained on a standard diet and overseen daily by veterinary staff accredited by the American Association for Accreditation of Laboratory Animal Care.

### Patient samples and sample collection

Deidentified pancreatic tumor tissue from patients with PDAC (*n* = 3) undergoing pancreaticoduodenectomy was used for Ig localization studies. Deidentified tissue was received from surgical pathology at UAB after specimen collection by a pathologist for diagnosis and staging of disease. Studies were conducted with an IRB-approved protocol where preoperative patients gave consent for their remnant, deidentified pancreatic tissue to be made available for tissue banking and research purposes.

### PCR genotyping

DNA was isolated from tail snips from newly weened pups after overnight digestion with proteinase K. The following primer sets were used: Cre F By1208: 5′-CCG TTT GCC GGT CGT GGG CGG CAT GG-3′; Cre R By1209: 5′-CGC GCG GCT CCG ACA CGG GCA CTG-3′; Kras: Y116: 5′-TCC GAA TTC AGT GAC TAC AGA TG-3′; Y117: 5′-CTA GCC ACC ATG GCT TGA GT-3′; Y118: 5′-ATG TCT TTC CCC AGC ACA GT-3′; Tp53 T008: 5′-CAC AAA AAC AGG TTA AAC CCA G-3′; Tp53 T009:5′-AGC ACA TAG GAG GCA GAG AC-3′; MS-8856: 5′-TGG AAC TCC GGA GAG ACC TA-3′; 8857: 5′-GAG GCA AGT ATG CAG GGT GT-3′; 01MR4074: 5′-AGG CTC AGG AGG AAG AGG AC-3′; AID M11: 5′-CTT GGG TGG AGA GGC TAT TCG GCT-3′; AID R1: 5′-CCA GGC TTT GAA AGT TCT TTC ACG-3′; 227: 5′-CAA CGT GGC GTC CAA ACA GGC ACT TCC G-3′.

### Histology and IHC

Pancreas/tumor, whole lung, and liver specimens were fixed overnight in 10% neutral-buffered formalin (Fisher Scientific) before being processed/embedded in paraffin blocks. Unstained sections, 5 μM thickness, were generated for downstream applications (H&E, Masson’s trichrome, IHC). For assessment of acinar-to-ductal metaplasia (ADM), intraepithelial neoplastic stages 1–3 (mPanIn1-3), pancreatic ductal tumor formation (PDAC), morphology/mitotic figures, metastases (lung and liver), necrosis, and extent of fibrosis (Masson’s trichrome) samples were evaluated in blinded fashion by a board-certified veterinary anatomic pathologist. For quantification of ADM, mPanIn1-3, and tumor morphology, 10 random 200x magnification images of H&E-stained lesion areas were recorded (Nikon Eclipse Cii and Nikon Elements D software), and then premalignant lesions and tumor morphologies were evaluated in a blinded manner. The total number of duct subtypes (normal, mPanIn1, mPanIn2, and mPanIn3) were counted and then averaged as the number of each duct/20x field of magnification. The total number of animals/genotype were pooled according to pre- and neoplastic stages/animal, and the mean ± SEM was graphed.

Evaluation of immune cell infiltrate, tumor type (epithelial-keratin 17/19 and E-cadherin staining versus mesenchymal-vimentin and αSMA staining), and stroma (podoplanin, αSMA, and vimentin) were evaluated using validated antibodies for formalin-fixed/paraffin-embedded specimens as outlined in Supplemental Table 1. Briefly, 5 μM unstained sections were deparaffinized, underwent citrate pH 6.0–based antigen retrieval (with exception of CD3/CD4/CD8, where Tris-EDTA pH 9.0 antigen retrieval was used), blocked with 5% BSA, incubated with the corresponding sera (rat or rabbit) based on the origin of the primary antibody for 1 hour at room temperature, washed, and then incubated overnight with primary antibody. Signal was then visualized using Vectastain ABC kit (rat and rabbit, Vector Labs) followed by development with DAB chromogen substrate, the hematoxylin counterstain, and cover slipping.

### Vascular perfusion studies

Five-month-old KPC-WT or KPC-μSAID mice were injected with 1 mg of 2,000 kDa FITC-dextran (Sigma-Aldrich) i.v. through the tail vein. After 5 minutes, mice were euthanized and the pancreas and spleen were harvested for downstream immune-fluorescence analysis of FITC-dextran and CD31 colocalization.

### Immune-fluorescence analyses

Pancreas/tumor and spleen were either fixed overnight in 4% paraformaldehyde (Thermo Fisher), transferred to 20% sucrose and incubated at 4°C for 48–72 hours, then embedded in OCT and flash-frozen in liquid nitrogen; or directly embedded in OCT and flash-frozen in liquid nitrogen (FITC-dextran perfusion study). From OCT-embedded blocks, 5 μM unstained sections were cut for IF. Evaluation of fibroblast, epithelium, immune cell subsets, FITC-dextran perfusion, and antibody deposition within mouse or human PDAC specimens was done using direct fluorochrome-conjugated antibodies as listed in Supplemental Table 1. Briefly, unstained sections from 4% PFA-fixed specimens were air-dried, blocked with 1% goat serum in PBS/0.1% Tween-20 for 1 hour, and then stained with primary fluorochrome-conjugated antibodies. For specimens directly embedded in OCT and flash frozen, slides were incubated in ice-cold acetone for 5 minutes prior to staining as outlined above.

### Quantification of IHC and IF data

All analyses were done in blinded assessment by a board-certified veterinary anatomic pathologist. Between 5–20 areas were imaged depending on the application (IF: 5, IHC: 5–10, PSR+trichrome: 5–10, and H&E: 10–20 images) and available amount of tissue on the slide. Individual random, 20x images were recorded on a Nikon Eclipse Ci microscope, using Nikon Elements D software (version 5.11.03 64-bit) then analyzed using ImageJ, Fiji Software (version 2.9.0 / September 14, 2022, Windows 64-bit). For IF analyses, 10x images were recorded on a Leica DMi8 microscope (Leica Microsystems) using fluorochrome-conjugated antibodies specific for EpCAM (AF488), podoplanin (AF647), F4/80 (AF647), CD31 (PE), or IgG (AF555). IF images were acquired with a C5810 series digital color camera (Hamamatsu Photonic System). Images were processed with Adobe Photoshop and IP LAB Spectrum software (Signal Analytics Software). For analysis, images were placed in RGB stack, gray-scaled then thresholded in red (IgG-AF555, CD31-PE), green (podoplanin, F4/80, FITC-dextran), or blue pseudo-colored channels (DAPI), which provided the best contrast to quantify area and number of positive particles. The histogram was adjusted to highlight positive areas of staining and compared with the color image. Regions of epithelial and stromal staining (IgG and IgM) were manually identified within regions of interest and then measured to calculate the percent area of IgG or IgM staining for these regions using ImageJ. To confirm colocalization of IgG antibodies with either EpCAM, podoplanin, or F4/80; IgG was merged with either EPCAM, podoplanin, or F4/80 (pseudo-colored green) to generate a red/green composite image in RGB scale. Composite images were then color-thresholded to highlight regions of yellow coloration and the area of yellow was then measured in ImageJ (NIH). The percent colocalization was calculated as follows: the area of yellow coloration (IgG-marker overlap) for a marker of interest was divided by total area of marker expression (EpCAM, podoplanin, or F4/80) before being multiplied by 100 to generate the percent colocalization. For evaluation of FITC-dextran pancreas perfusion, regions of neoplasia were imaged, and the percent area of FITC-dextran and percent area of CD31 were calculated using ImageJ before being divided by each other to generate ratios of percent FITC-dextran/percent CD31 areas. For assessment of cell numbers with IF and IHC markers, individual cells were then counted using the analyze-particles function. Numbers of cells or percent area of positive staining were enumerated for three to five 20x fields and were then averaged per specimen and reported as raw data.

### ELISA

#### Immunoglobulin.

Serum and tumor lysates were used to quantify immunoglobulin G, G1, G2b, G3, A, M, and E as follows: (a) 96-well half-area ELISA plates (Thermo Fisher) were coated overnight with polyclonal goat anti-mouse antibodies specific for IgG, IgG1, IgG2b, IgG3, IgA, IgM, and IgE (Southern Biotech) at a concentration of 1 mg/mL in PBS, (b) plates were washed in PBS, blocked in a 1% BSA solution for 1 hour at 4°C, (c) sera were diluted between 1/50 to 1/4,000 depending on the Ig isotype being measured (IgE-1/50, IgA-1/250, IgM-1/500, IgG isotypes 1/1,000–1/4,000) and then incubated at 37°C for 2 hours, (d) plates were washed and developed with goat anti-mouse alkaline phosphatase secondary-specific antibodies (Southern Biotech) at a 1/2,000 dilution for 1 hour at 37°C, (e) plates were washed and then developed with pNPP liquid substrate for 10–30 minutes at room temperature, and (f) the reaction was quenched with stop solution and absorbance measured at λ405 and λ450 using a SpectraMax M5 microplate reader (Molecular Devices LLC). For tissue lysates, 100 mg of pancreatic tissue was sonicated in 1 mL M-PER buffer (Thermo Fisher) containing 1x HALT protease and phosphatase inhibitor cocktail (Thermo Fisher). Lysates were cleared of debris by microfuge centrifugation for 1 minute followed by flash freezing of the supernatant or immediate evaluation by ELISA.

#### Procollagen 1.

Tissue lysates processed as described above were used for evaluation of procollagen 1 levels using a Procollagen 1 ELISA kit (Abcam). Tissue lysates were diluted at 1/100 for normal pancreas from Pdx1-Cre-WT mice and 1/1,000 for pancreas lysates from KC- and tumor-bearing KPC-WT mice. ELISAs were carried out following manufacturer’s instructions.

### Flow cytometry

Prior to collection of pancreas for flow cytometry, mice were perfused with 25 mL ice-cold PBS. For FACS, the tail of the pancreas was collected in an attempt to avoid contamination with draining (pancreatic, hepatic, duodenal) lymph nodes, which can become encapsulated by ECM and locally invaded by tumor. Specimens were digested in a 1 mg/mL collagenase IV, 1 mg/mL Liberase (Millipore Sigma), and 0.1 mg/mL DNase I solution (Worthington Biochemical) in HBSS for 45 minutes at 37°C with intermittent shaking every 5–10 minutes. Samples were gently pipetted to break up aggregates then diluted with RPMI containing 10% FBS and filtered through a 70 μM cell strainer. Cells were pelleted by centrifugation at 300*g*, resuspended in PBS plus 2% FBS, and then refiltered prior to cell counting and flow cytometry. Cells were labeled with primary fluorochrome-conjugated antibodies and a live/dead stain (Supplemental Table 2) for 30–60 minutes at 4°C, washed, and then resuspended in PBS with 2% FBS. Cytokine expression from single-cell suspensions of spleen and tumor was quantified by (a) plating at a concentration of 1 x 10^6^ cells in 1 mL of RP-10 media in 96-well round-bottom plates followed by (b), a 5-hour stimulation at 37°C using a 1x solution of a Cell Activation Cocktail containing PMA, ionomycin, and 5 μg/mL Brefeldin A (BioLegend). After cell-surface staining, cells were fixed in 4% paraformaldehyde for 45 minutes at 4°C then washed with 1x Perm/Wash (Becton-Dickinson, 554723). For intracellular staining in tumor and spleen samples, cells were stained with antibody cocktail-specific intracellular cytokines (IFN-γ) in Perm/Wash solution for 60 minutes at 4°C. For quantification of Foxp3 expression, a commercially available Foxp3/Transcription Factor Staining kit (Thermo Fisher) was utilized prior to intra-nuclear staining for Foxp3. Data were acquired on a Symphony A5 flow cytometer, with analysis performed using FlowJo version 10.7.2.

### Statistics

Sample sizes were determined based on pilot studies to achieve 80% statistical power with a 95% CI. GraphPad Prism (version 9.1.2) was used for statistical analyses, and graphical representation with data presented as either means ± SD or SEM. Two-tailed Student’s *t* test and 2-way ANOVA with multiple corrections (Tukey’s or Šídák’s test for multiple comparisons) were performed for determining the statistical significance between groups. Log-rank and Gehan-Breslow-Wilcoxon test were used to evaluate statistical significance, along with calculation of median survival times. Results were considered statistically significant at *P* values of *P* < 0.05.

### Study approval

All animal studies were performed under IACUC-approved protocols according to the University of Alabama at Brimingham’s IACUC guidelines.

### Data availability

Raw FACS, ELISA, IF/IHC/H&E/PSR/trichrome images, survival data, morphologic characterization (tumor grade, type, mitotic figures, etc.) are provided in the XLS document for all data represented as mean ± SD/SEM with the appropriate statistical testing listed in each tab. Values for all data points in graphs are reported in the Supporting Data Values file.

## Author contributions

JBF and CAK conceived the project and experimental design. JBF, SS, CAK, CH, and MK preformed experiments/acquired raw data. Blinded assessments on histopathology lesion spectra were performed by JBF. BJR and SAD were critical for acquiring patient tumor samples and pathologic assessment of patients. SS, KK, DKC, JLC, and CC assisted with experimental design, data interpretation and paper edits.

## Conflict of interest

The authors have declared that no conflict of interest exists.

## Funding support

This work is in part the result of NIH funding and is subject to the NIH Public Access Policy where the results are publicly available in PubMed Central.

- Richard A. Elkus Foundation for Pancreatic Cancer Research and a pilot grant award to JBF and CAK from the UAB O’Neil Comprehensive Cancer Center Support grant (P30 CA013148).

## Supplementary Material

Supplemental data

Supporting data values

## Version 1 05/11/2026

In-Press Preview

## Version 2 07/08/2026

Electronic publication
