# Supplementary material for: Antibody subclass deficiency accelerates tumorigenesis in genetically engineered mouse models of pancreatic cancer
Source: JCI Insight. 2026 May 5;11(13):e198489. doi: 10.1172/jci.insight.198489 (PMC13460815; doi:10.1172/jci.insight.198489)
Supplement: Supplementary file 1 [file jciinsight-11-198489-s191.pdf]

Supplemental Figures and Legends

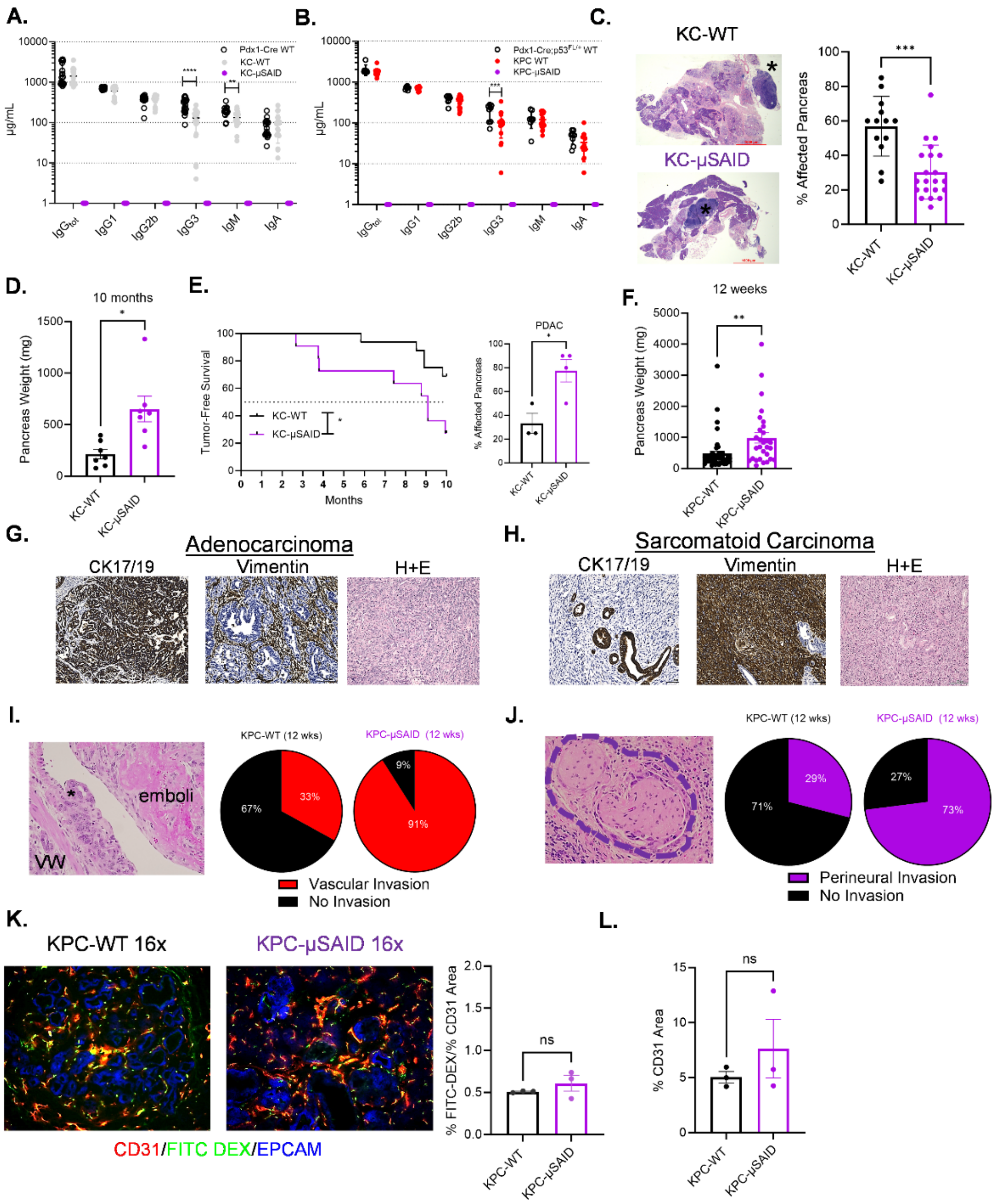

Supplemental Figure 1. Antibody deficiency enhances metastatic phenotypes in KC-μSAID pancreata. (**A**) Levels of Ig isotype antibodies in blood of 5-month-old Pdx1-Cre WT, KC-WT, and KC-μSAID mice based on ELISA, *n* = 5 - 15 mice/genotype, mean +/- SD, ***P* < 0.01, *****P* < 0.0001; 2-way ANOVA with Tukey's multiple comparison test. (**B**) Levels of Ig isotype antibodies in peripheral blood of 5-month-old Pdx1-Cre;53$^{FL/+}$ WT, KPC-WT, KPC-μSAID mice, *n* = 5 - 15 mice/genotype, mean +/- SD, ****P* < 0.001; 2-way ANOVA with Tukey's multiple comparison test. (**C**) Left panel: Low magnification images of pancreas from 5-month-old KC-WT (top) and KC-μSAID mice. The extent of pancreas pathology was evaluated in a semi-quantitative, blinded manner by a board-certified veterinary anatomic pathologist (JBF). Right: The % normal and affected regions of pancreas from KC-WT (*n* = 13) and KC-μSAID (*n* = 21) mice, mean +/- SD, ****P* < 0.001; Welch's *t* test. (**D**) Pancreas weights in 10-month-old KC-WT (*n* = 7) and KC-μSAID mice (*n* = 6), mean +/- SD, **P* < 0.05; Welch's *t* test. (**E**) Left panel: Kaplan-Meier survival curve of KC-WT and KC-μSAID mice, *n* = 11 - 16 mice/genotype. **P* < 0.05; log-rank test. Right panel: Assessment of % affected pancreas in available H+E-stained specimens from 10-month-old KC-WT (*n* = 3) and KC-μSAID (*n* = 5) mice. **P* < 0.05; Welch's *t* test. (**F**) Pancreas weights in 3-month-old KPC-WT (*n* = 44) and KPC-μSAID mice (*n* = 29). Data is represented as mean +/- SD, ***P* < 0.01; Welch's *t* test. (**G**) Representative IHC for CK17/19 and Vimentin in adenocarcinoma and (**H**) sarcomatoid carcinoma of the pancreas in KPC mice. (**I-J**) The extent of vascular (**I**) and perineural (**J**) invasion was examined in 12-week-old KPC-WT and KPC-μSAID mice. *Is indicative of region of tumor cell invasion (**K**) Vascular permeability was evaluated by IV injection of 1 mg of FITC-dextran (2,000 kDa) in KPC-WT (*n* = 3) and KPC-μSAID (*n* = 3) mice. FITC-dextran (green), CD31 (red), and EpCAM (blue) are represented in the images on the left panel. The ratio of % FITC-dextran: % CD31 area is graphed in the right panel, mean +/- SD, ns = non-significant; Welch's *t* test. (**L**) The area of CD31^{+} vascular staining was

determined in neoplastic regions in KPC-WT and KPC-µSAID mice, ns = non-significant; Welch's *t* test.

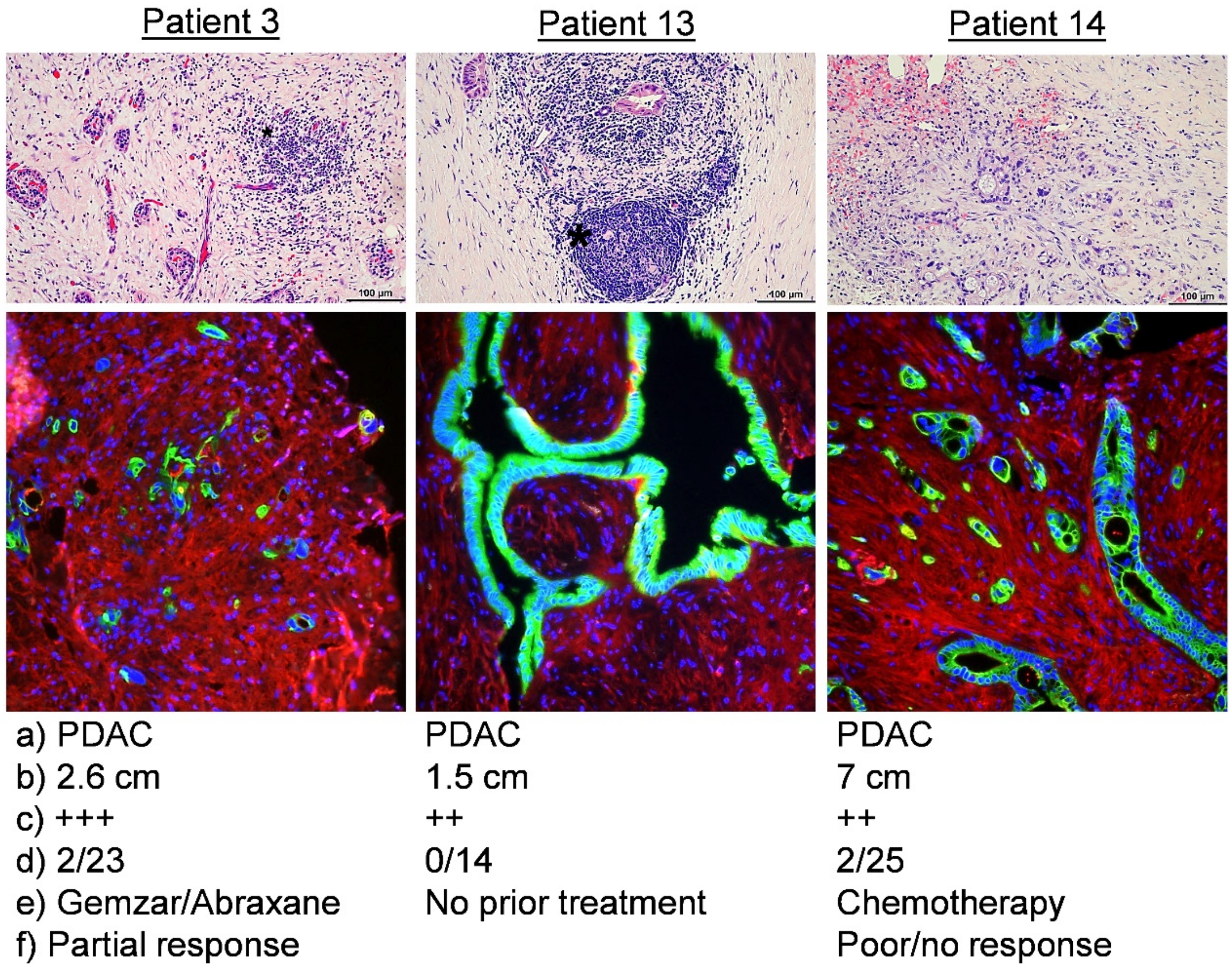

Supplemental Figure 2. IgG and IgM binding to tumor epithelium in human PDAC patient samples. Representative H+E (top panel) and immunofluorescent images of human PDAC tumor tissue stained with goat anti-human IgG-AF555, rat anti-human CK7-AF488, and DAPI [(a) diagnoses, (b) tumor size, (c) extent of ECM deposition, (d) number of lymph nodes containing tumor, (e) treatment prior to surgical resection, and (f) patient response to treatment are indicated below IF images]. TLS location noted in patient 13 H+E noted adjacent to asterisk.

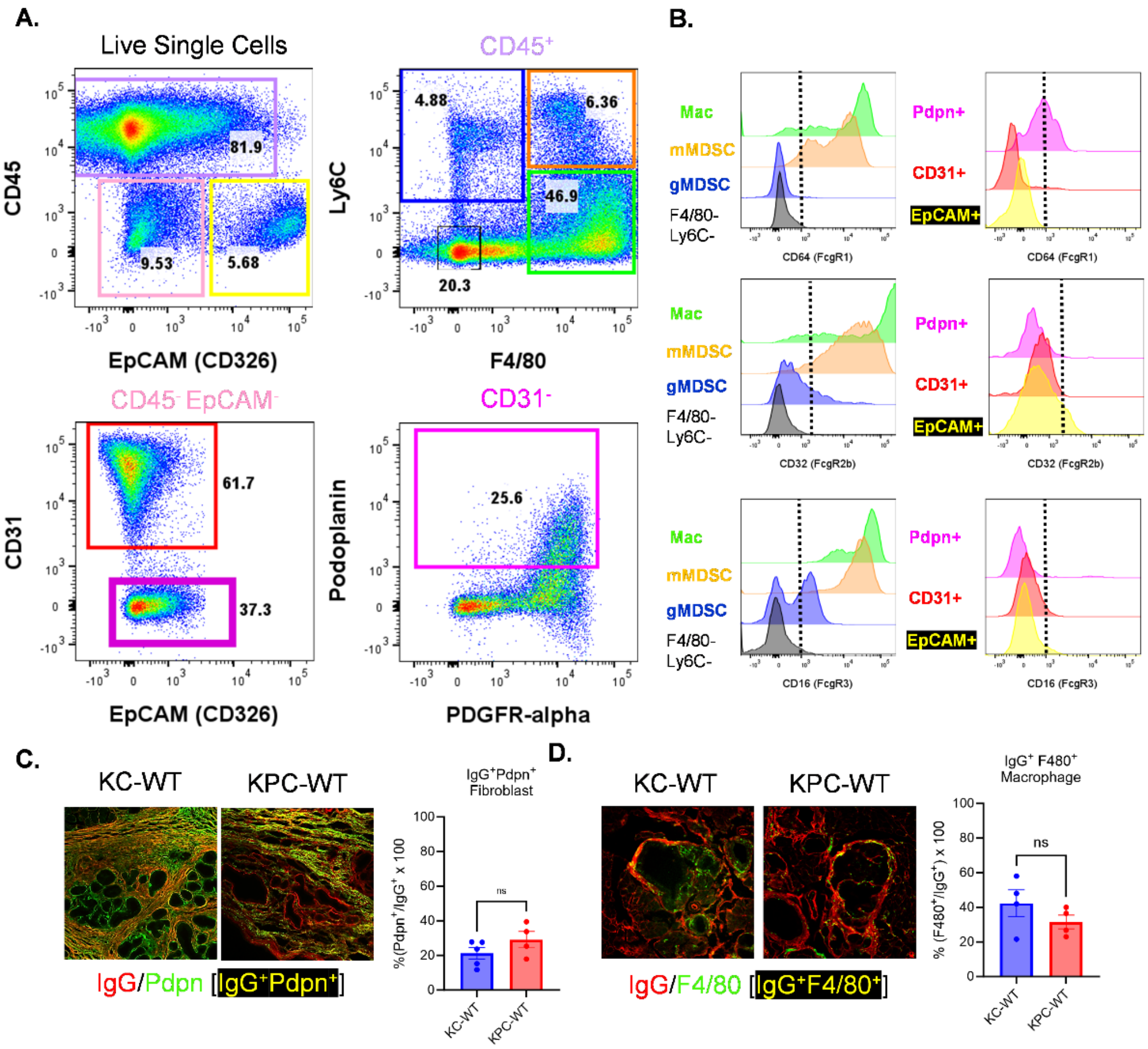

Supplemental Figure 3. IgG deposition and FcγR expression on stromal and myeloid cells in the pancreatic microenvironment. (**A**) Cell gating scheme for CD45$^+$ subsets including CD45$^+$Ly6C$^-$F4/80$^-$, CD45$^+$Ly6C^{int}F4/80$^-$(gMDSC), CD45$^+$Ly6C^{hi}F4/80^{int} (mMDSC), CD45$^+$Ly6C^{lo}F4/80$^+$ (macrophage), CD45$^-$EpCAM$^+$ epithelia, and CD45$^-$EPCAM$^-$ mesenchymal cells including CD31$^+$ endothelium and CD31$^-$ Podoplanin$^+$ CAFs. (**B**) Representative staggered histograms depicting FcγR1 (CD64), FcγR2b (CD32), and FcγR3 (CD16) on the afore-mentioned subsets in panel A. (**C**) Right panel: Representative IF images of IgG and podoplanin expression. Left panel: The % colorimetric overlap for IgG proximal to podoplanin$^+$ cells was quantified in antibody-sufficient, KC-WT and KPC-WT mice (*n* = 4 - 5 mice/group), mean +/- SD, ns = non-significant; Welch's *t* test. (**D**) Right panel: Representative

IF images of IgG and F4/80 expression. Left panel: The % colorimetric overlap for IgG proximal to F/480$^+$ cells was quantified in antibody-sufficient, KC-WT and KPC-WT mice (n = 4 mice/group), mean +/- SD, ns = non-significant; Welch's t test.

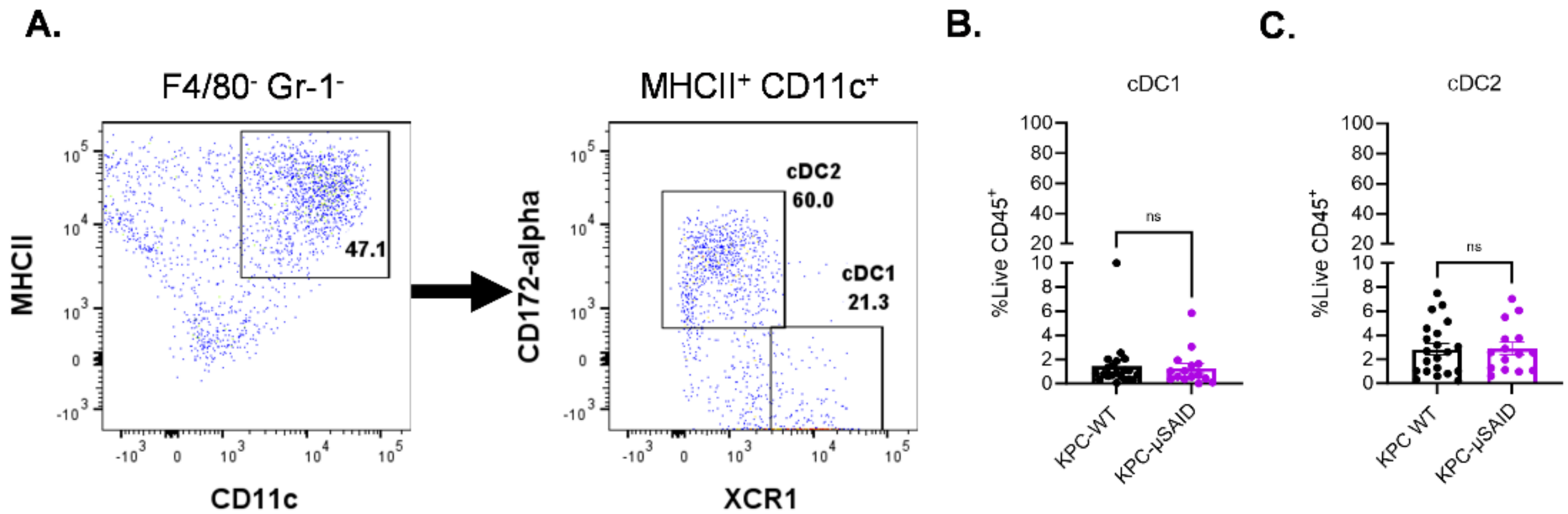

Supplemental Figure 4. Multiparameter flow cytometric analysis of dendritic cell subsets isolated from the pancreas of perfused mice. (**A**) Identification of dendritic cell subsets in the $CD11b^{+/-}F4/80^{-}Gr\text{-}1^{-}$ subset using MHCII and CD11c followed by XCR1 (cDC1) and CD172α (cDC2). (**B-C**) Percentages of tumor-infiltrating $CD11b^{-}CD172\alpha^{-}XCR1^{+}$ cDC1 (**B**), and $CD11b^{+}CD172\alpha^{+}$ cDC2 (**C**), in 12-week-old KPC-WT vs. KPC-μSAID TME (*n* = 15 - 20 mice/group), mean +/- SEM, ns = non-significant; Welch's *t* test.

Supplemental Table 1. Immunohistochemistry Primary Antibodies

Antigen	Clone	Vendor	Antigen Retrieval	Dilution	RRID #
CD3	CD3-12; rat IgG1	Abcam	TRIS-EDTA pH 9	1/400	AB_2889189
CD4	4SM95; rat IgG1	Invitrogen	TRIS-EDTA pH 9	1/400	AB_2573008
CD8	4SM16; rat IgG2a	Invitrogen	TRIS-EDTA pH 9	1/400	AB_2637159
Foxp3	FJK-16s; rat IgG2a	Invitrogen	Citrate pH 6	1/200	AB_467576
F4/80	CI:A3-1; rat IgG2b	BioRad	Citrate pH 6	1/400	AB_323279
MPO	2D4; Mouse IgG1	Abcam	Citrate pH 6	1/400	AB_2050022
B220	RA3-6B2; rat IgG2a	BD	Citrate pH 6	1/200	AB_394614
Keratin 17/19	D32D9; rabbit mAb	Cell Signal	Citrate pH 6	1/1000	AB_2797912
Podoplanin	Pmab-1;rat IgG2a	BioLegend	n/a	1/200	AB_2750408
CD326	G8.8;rat IgG2a	BioLegend	n/a	1/200	AB_1134099
IgA	Polyclonal	SBA	n/a	1/500	AB_2794378
IgG	Polyclonal	SBA	n/a	1/2000	AB_2794359
IgM	Polyclonal	SBA;1020-32	n/a	1/500	AB_2794223
F4/80	BM8	BioLegend	n/a	1/200	AB_893480
CD31	390	BioLegend	n/a	1/200	AB_312903

Supplemental Table 2. FACS Antibody List

Antibody	Supplier	Clone	Dilution	Cat	RRID
NK 1.1 FITC	BioLegend	PK136	1-100	108705	AB_ 313392
PD-1 FITC	BioLegend	29F.1A12	1-200	135214	AB_ 10680238
CD45 PE	BD	30-F11	1-800	561087	AB_ 394611
F4/80 PE	BioLegend	BM8	1-800	123110	AB_ 893486
TNFα PE	BioLegend	MP6-XT22	1-200	517008	AB_ 315426
Gr-1 PE Cy7	BioLegend	RB6-8C5	1-1000	108416	AB_ 313380
Granzyme B PE Cy7	BioLegend	GB11	5 uL/10 cells	372214	AB_ 2728380
Ly6 C PE Cy7	BioLegend	HK1.4	1-3000	128017	AB_ 1732082
CD8 PE Cy7	BioLegend	53-7.3	1-400	100622	AB_ 2562772
CD11b PE 594	BioLegend	M1/70	1-3000	101255	AB_ 2563647
IFNγ PE 594	BioLegend	XMG1.2	1-1000	505846	AB_ 2563979
CD45 PerCP	BioLegend	30-F11	1-800	103130	AB_ 893339
TCRγδ PerCP Cy 5.5	BioLegend	GL3	1-200	118117	AB_ 10612572
Foxp3 APC	Invitrogen	FJK-16s	1-200	17-5773-82	AB_ 469457
CD11c APC	BioLegend	N418	1-400	117309	AB_ 313778
CD172α Alexa Flour 700	BioLegend	P84	1-1000	144022	AB_ 2650812
CD62L APC Cy7	BioLegend	MEL-14	1-800	104428	AB_ 830798
Podoplanin APC Cy7	BioLegend	8.1.1	1-200	127418	AB_2629804
XCR1 BV 421	BioLegend	ZET	1-1000	148216	AB_ 2565230
CD11b Pacific Blue	BioLegend	M1/70	1-1000	101224	AB_ 755985
Live Dead Aqua	Invitrogen	n/a	1-500	L34966	
PD-L1 BV605	BioLegend	MIH5	1-400	153606	AB_ 2814056
MHCII BV 650	BioLegend	M5/114/15.2	1-800	107641	AB_ 2565975
CD4 BV 650	BioLegend	GK1.5	1-800	100469	AB_2783035
CD31 BV785	BioLegend	390	1-800	102435	AB_2810334
CD44 BUV 7 3 7	BD	IM7	1-800	612799	AB 2870126
Fc Block CD16/CD32	BioLegend	93	1-500	101320	AB 10766423
